# Beneath the Surface: Unveiling Idiopathic Granulomatous Mastitis Within a Masquerading Breast Lump

**DOI:** 10.7759/cureus.92678

**Published:** 2025-09-18

**Authors:** Nitesh Kumar, Deepak Pankaj, Saumya Sinha, Pooja Kumari, Smita Pawar, Vibhuti Bhushan, Zeenat S Imam, Sweta Muni, Vishwapal Vishwendu

**Affiliations:** 1 General Surgery, Indira Gandhi Institute of Medical Sciences, Patna, IND; 2 Pathology, Indira Gandhi Institute of Medical Sciences, Patna, IND; 3 Microbiology, Indira Gandhi Institute of Medical Sciences, Patna, IND

**Keywords:** granuloma, idiopathic granulomatous mastitis, inflammatory breast disease, methotrexate, steroids

## Abstract

Background

Idiopathic granulomatous mastitis (IGM) is a rare, benign, chronic inflammatory breast disease of uncertain etiology that often mimics malignancy, thus posing significant diagnostic and therapeutic challenges.

Aim

To evaluate the clinical profile, diagnostic pathways, and management outcomes of IGM in women in Eastern India through a prospective cohort study.

Methods

This prospective, observational study was conducted from July 2024 to June 2025 at a tertiary care center in Eastern India. Fifty-four female patients presenting with breast complaints suggestive of IGM were recruited. Patients underwent detailed evaluation, including clinical examination, imaging, core needle biopsy, microbiological studies, and individualized management protocols. Follow-up was conducted at one, three, and six months.

Results

The mean age was 36.1 ± 7.3 years; all 54 (100%) participants were parous. The most common presenting symptom was a palpable breast lump in 48 (88.9%) patients, followed by pain in 38 (70.4%). Bilateral involvement was observed in seven (13.0%). Radiologically, lesions were non-specific, commonly appearing as heteroechoic/hypoechoic masses or abscesses. Histopathology confirmed non-caseating granulomatous mastitis in all 54 (100%) cases. Microbiological cultures were negative in 49 (90.7%). Treatment was individualized: 25 (46.3%) required surgery, and 29 (53.7%) received medical management (steroids, methotrexate, antibiotics). At six months, 34 (63.0%) achieved partial remission, eight (14.8%) attained complete remission, 12 (22.2%) had a chronic/progressive course, and four (7.4%) experienced recurrence.

Conclusions

IGM remains a clinical diagnostic challenge in Indian practice. Early histopathological confirmation and targeted medical management, especially steroid-based therapy, are essential to minimize morbidity. Multidisciplinary individualized approaches are critical for optimal outcomes; high recurrence rates after surgery highlight the need for avoidant surgical overtreatment.

## Introduction

Idiopathic granulomatous mastitis (IGM) is an increasingly recognized, benign yet locally aggressive inflammatory condition of the breast first described by Kessler and Wolloch in 1972 [1]. The condition predominantly affects young to middle-aged women, frequently those of reproductive age, and is particularly prevalent among parous and lactating women [2]. IGM commonly simulates infectious or malignant breast disorders, which contribute to its diagnostic complexity. Despite its benign histology, IGM can follow a chronic, relapsing course with a significant impact on quality of life and breast cosmesis [3].

While global literature on IGM is growing, it remains underreported from low- and middle-income countries, including India. Here, the challenge is compounded by overlapping presentations with tuberculosis (TB) and limited access to advanced diagnostic modalities [4,5]. Despite being benign, its management remains controversial. Reported modalities range from observation and antibiotics to corticosteroids, immunosuppressants, and surgery. Al-Khaffaf et al. highlighted heterogeneity in treatment and frequent recurrence after surgery [6], while Donn et al. stressed the need for recognition to avoid overtreatment [7]. Shin et al. questioned the necessity of surgical excision [8], and Gurleyik et al. showed both medical and surgical options carry risks of recurrence [9].

The aim of this study is to prospectively document the clinical spectrum, diagnostic strategies, and therapeutic outcomes in a series of Indian women diagnosed with IGM at a single tertiary care center, and to review the contemporary evidence to guide best practice.

## Materials and methods

Study design and setting

A prospective, observational study was conducted at the Department of General Surgery, Indira Gandhi Institute of Medical Sciences (IGIMS), Patna, India, from July 2024 through June 2025. Ethical approval was obtained from the Institutional Ethics Committee, IGIMS (227/IEC/IGIMS/2024). Written informed consent was procured from all participants.

Inclusion and exclusion criteria

Female patients aged 16-60 years with newly diagnosed, histopathologically confirmed granulomatous mastitis were included in the study, provided no alternative etiology was identified on special stains or cultures (idiopathic variety). Female patients with a definitive diagnosis of tuberculosis, sarcoidosis, fungal infection, or malignancy, as well as individuals lost to follow-up or with incomplete records, were excluded from the analysis.

Sample size

The sample size was calculated based on a presumed prevalence of IGM of 2.4 per 100,000 population, with 95% confidence and 5% precision, yielding a minimum of 54 cases. Accordingly, all eligible patients presenting during the study period were included [10].

Clinical and diagnostic workup

A detailed clinical history was obtained for each patient, covering demographics, parity, breastfeeding duration and exposure, use of oral contraceptives (OCP), personal or family history of tuberculosis or autoimmune disorders, and any prior breast surgery. Clinical examination assessed the site, size, and characteristics of breast masses, as well as local signs such as erythema, abscesses, or sinus tracts. Lymph node status, bilaterality, and skin or nipple involvement were also carefully documented.

Laboratory and imaging evaluation included ultrasonography for characterization of masses, abscesses, or sinus tracts, while mammography was performed in women over 40 years of age. Core needle biopsy specimens were obtained from all patients for histopathological evaluation. Microbiological analysis of pus or tissue samples was performed for aerobic and anaerobic bacteria, acid-fast bacilli, and fungi. Histopathological confirmation required the presence of non-caseating granulomas with giant cells centered around lobules, and all samples were negative for acid-fast bacilli (AFB), fungi, or malignancy.

Management

Treatment strategies were individualized according to clinical severity and patient preference. Medical therapy comprised oral corticosteroids (prednisolone 0.5-1 mg/kg), with or without methotrexate (10-15 mg/week), and antibiotics were added when secondary infection was suspected. Surgical interventions included incision and drainage (I&D) for abscesses or collections, or wide local excision (WLE) for persistent or recurrent masses and non-responsive cases. Antitubercular therapy (ATT) was reserved strictly for patients with high clinical suspicion and confirmed evidence of tuberculosis. All patients receiving medical therapy were closely monitored for drug-related adverse effects.

Follow-up and outcome definitions

Patients were followed up at one, three, and six months after initiation of therapy to assess clinical resolution, persistence, relapse, or complications. Complete remission (CR) was defined as total resolution of the mass and symptoms at six months. Partial remission (PR) refers to more than a 50% reduction in size or symptoms without new lesion development. A progressive or chronic disease course (PD) was defined as persistence of symptoms beyond six months or the appearance of new or worsening lesions.

## Results

In our cohort of 54 patients, the mean age was 36.1 years, and all 54 (100%) were parous, with an average lactation duration of 20.4 months. The predominant presenting symptom was a palpable lump in 48 (88.9%) patients, followed by pain in 38 (70.4%), while seven (13.0%) demonstrated bilateral involvement (Table 1).

**Table 1:** Demographic and clinical features of patients Percentages are calculated based on the total cohort size (n = 54) OCP: oral contraceptives; TB: tuberculosis

Parameter	Number
Mean age (years)	36.1 ± 7.3
Parity	54 (100%)
Mean lactation (months)	20.4 ± 22.4
Lump	48 (88.9%)
Pain	38 (70.4%)
Skin changes (erythema, peeling)	15 (27.8%)
Nipple discharge	3 (5.5%)
Bilateral involvement	7 (3.0%)
Previous breast surgery	3 (5.5%)
OCP use	7 (13.0%)
Personal/family TB history	3 (5.5%)

Radiological findings were largely nonspecific, with ultrasonography showing abscesses or solid lesions in 33 (78.6%) of 42 evaluated cases, mammography (in 11 women >40 years) revealing only nonspecific shadows, and histopathology confirming non-caseating granulomas in all 54 (100%) patients. Microbiological cultures were negative in 49 (90.7%) (Table 2).

**Table 2:** Integrated diagnostic findings Percentages are calculated based on the total cohort size (n = 54) TB: tuberculosis

Modality	Findings	Number
Ultrasonography (USG)	Mass lesion	42 (77.8%)
Mammography	Non-specific lesion	11 (20.4%)
Histopathology	Non-caseating granuloma	54 (100%)
Microbial culture (excluding TB/fungi)	Negative	49 (90.7%)

Treatment was individualized, with 25 (46.3%) managed medically (steroids ± methotrexate ± antibiotics), 25 (46.3%) undergoing combined surgical and medical approaches, and four (7.4%) receiving antitubercular therapy. At six months, remission (complete or partial) was achieved in 42 (77.8%) patients, including eight (14.8%) with complete remission and 34 (63.0%) with partial remission. Progression or relapse occurred in 12 (22.2%), with higher rates in the surgical group (8/25; 32.0%) compared to the medical group (4/25; 16.0%) (Table 3).

**Table 3:** Treatment Modalities and Outcomes Complete remission was higher in the medical group (16.0% vs. 4.0%), though not statistically significant (p = 0.349); progression/relapse was more frequent after surgery (32.0% vs. 16.0%), also not statistically significant (p = 0.281) MTX: methotrexate; I&D: incision and drainage; WLE: wide local excision; Rx: treatment; ATT: antitubercular therapy; TB: tuberculosis

Treatment modality	n (%)	Partial Remission n (%)	Complete remission n (%)	Progression/relapse n (%)
Medical (steroids ± MTX ± antibiotics)	25 (46.3%)	17 (68.0%)	4 (16.0%)	4 (16.0%)
Surgery + medical (I&D/WLE + Rx)	25 (46.3%)	16 (64.0%)	1 (4.0%)	8 (32.0%)
ATT for TB-suspect only	4 (7.4%)	1 (25.0%)	3 (75.0%)	0 (0%)
Total	54 (100%)	34 (63.0%)	8 (14.8%)	12 (22.2%)

## Discussion

IGM is a benign breast condition for which no definitive or universally accepted treatment protocol currently exists [11]. The mean age in our cohort was 36.1 years, and all 54 (100%) women were parous. The mean duration of lactation was 20.4 months, supporting the established association between prior lactation and IGM development [12,13].

Skin involvement and non-specific signs such as erythema, induration, peeling, and occasionally sinus formation were present in 15 (27.8%) patients, which is consistent with literature from the Middle East and South Asia, where late presentations are frequent [13]. Bilateral disease, though described as rare (<10% in the literature), was seen in seven (13.0%) patients in our series, reaffirming more aggressive or neglected presentations in Indian settings [14].

Diagnostic work-up and challenges

While IGM is characterized by non-caseating lobulocentric granulomas on histology, the diagnosis remains challenging, particularly in regions like India, where TB, fungal infections, sarcoidosis, and breast malignancy are significant differential considerations [15]. Radiologically, ultrasonography and mammography often reveal only non-specific findings, such as hypoechoic or heteroechoic masses, abscesses, or ductal ectasia, making tissue biopsy mandatory. In our study, all 54 (100%) cases were ultimately confirmed with core needle biopsy, which demonstrated non-caseating granulomas, and special stains excluded TB and fungal disease [15].

Microbial cultures for pyogenic organisms and mycobacteria were negative in 49 (90.7%) patients, consistent with reports describing IGM as a sterile, idiopathic inflammation. Nonetheless, a potential role for subclinical pathogens, particularly *Corynebacterium kroppenstedtii*, has been postulated, although culture isolation of this organism is technically challenging and rarely performed in Indian practice [16,17].

Pathogenesis and risk factors

Several risk factors are hypothesized, including multiparity, breastfeeding, hormonal influences (e.g., OCP use), prior trauma, autoimmune predisposition, and possible infectious triggers [18,19]. The possible role of *Corynebacterium kroppenstedtii *and other subacute bacterial agents has been highlighted by recent molecular studies, notably in East Asian and European populations, but such data from the Indian setting are lacking [16,17,19].

Autoimmune or granulomatous processes targeting ductal epithelium of the breast, often precipitated by epithelial damage, are considered central to pathogenesis [20]. Associations with other autoimmune disorders, though uncommon in the present series (2/54; 3.7%), underscore the need for a high index of suspicion and careful systemic evaluation [19,20].

Treatment approaches

Medical Therapy

Corticosteroids remain the cornerstone of IGM management, with several studies confirming efficacy rates exceeding 70% [11,21,22]. In India, where infectious disease misdiagnoses and delayed presentations are routine, steroids are often used only after careful exclusion of TB [23]. Methotrexate and other immunosuppressants are advocated in steroid-refractory or relapsing cases, either as monotherapy or adjunct [22,24]. In our cohort, patients treated with corticosteroids (± methotrexate) achieved partial remission in 17/25 (68.0%) and complete remission in 4/25 (16.0%), with acceptable side-effect profiles like mild weight gain and gastritis. Antibiotics were used only for superadded infections and have no proven role as monotherapy [23].

Surgical Therapy

Surgical options ranged from simple aspiration/incision and drainage (I&D) for abscesses to wide local excision of involved tissue, but these must be approached cautiously to avoid excessive morbidity. Surgery alone carries a high risk of disease recurrence and poor cosmetic results, especially with aggressive resections [12,21]. In our study, 25/54 (46.3%) underwent some form of surgery, most often combined with medical therapy. Relapse was more common after surgery-even when followed by medical management-occurring in 8/25 (32.0%), compared with 4/25 (16.0%) in the medical-only group, underscoring current expert opinion against routine excision in favor of steroid-based regimens [21,25,26].

Tuberculosis Exclusion

Given TB’s high prevalence in India, empirical ATT is occasionally commenced in recalcitrant or suspicious cases, but this approach risks overtreatment. Only three (5.6%) cases in our series received ATT on a presumptive basis, two of which failed to resolve with standard therapy [15].

Outcomes and prognosis

At six months, overall remission (complete + partial) was achieved in 42/54 (77.8%) patients, comparable to rates in international literature [11,23,25]. However, recurrence and chronicity remain major problems: 12/54 (22.2%) patients had persistent or relapsing disease, with almost all recurrences seen in the surgically managed subgroup. Several meta-analyses have reported relapse rates between 10-30%, with risk factors including persistent disease activity, incomplete surgical excision, and absence of immunosuppression [11,21,25,26]. Prolonged follow-up is essential, as late relapses and scarring deformities can significantly impair quality of life [24].

Limitations

The principal limitations of our study are its single-center design and limited sample size, although our cohort is representative of the region. The follow-up period (six months) may underestimate relapses occurring after a year, a finding consistently emphasized in recent reviews. Moreover, standardized protocols for steroid and methotrexate dosing are lacking, reflecting real-world variation in clinical practice. Microbiological workup remains limited in many Indian centers, precluding pathogen discovery beyond readily culturable organisms.

## Conclusions

In this prospective cohort of 54 women with idiopathic granulomatous mastitis, overall remission was achieved in 77.8% at six months, while 22.2% experienced persistent or relapsing disease. Corticosteroid-based regimens (± methotrexate) showed better outcomes (84.0% remission, 16.0% recurrence) than surgery-inclusive approaches (68.0% remission, 32.0% recurrence). Early tissue diagnosis, exclusion of tuberculosis, and steroid-based therapy remain the most effective management strategy.
